# Differences in COVID-19 Fatality Rates among Ethnic Groups, Hawaii, USA, 2020–2022

**DOI:** 10.3201/eid3110.241070

**Published:** 2025-10

**Authors:** Gehan Devendra, Madeleine Chai, Eyrica Sumida, Richard Chen, Maan Gozun, Dominic Chow, F. DeWolfe Miller

**Affiliations:** University of Hawaii John A. Burns School of Medicine, Honolulu, Hawaii, USA (G. Devendra, M. Chai, E. Sumida, R. Chen, D. Chow, F. DeWolfe Miller); Queen’s Medical Center, Honolulu (G. Devendra, M. Gozun, D. Chow)

**Keywords:** COVID-19, respiratory infections, severe acute respiratory syndrome coronavirus 2, SARS-CoV-2, SARS, coronavirus disease, zoonoses, viruses, coronavirus, hospital, fatality rate, Asian American Pacific Islanders, vaccination, sequential organ failure assessment score, SOFA score, Hawaii, United States

## Abstract

Asian American, Native Hawaiian, and Pacific Islander populations have been underrepresented in research on COVID-19 outcomes. We conducted a cross-sectional study of 5,494 electronic medical records of patients in a large tertiary care health system in the ethnically diverse state of Hawaii, USA. We compared fatality rates for hospitalized patients on the basis of race or ethnicity, age, vaccination status, and sequential organ failure assessment (SOFA) score at admission. Fatality rates varied between racial and ethnic groups but were associated with increasing age across all groups. Fatality rates were closely associated with increasing SOFA score and were inversely associated with the number of COVID-19 vaccinations received. We found that Asian and Pacific Islander groups experienced higher rates of in-hospital death and that death was strongly associated with increased age and SOFA score and with <1 COVID-19 vaccination. Clinicians should be aware of these outcomes when treating COVID-19 patients from these ethnic groups.

The COVID-19 pandemic has been a major cause of illness and death globally, and the World Health Organization reported >770 million confirmed cases and >7 million deaths by February 2024 (*1*). Although differences in health outcomes and deaths among racial and ethnic groups have been a topic of research for many years, the COVID-19 pandemic magnified those differences. Several prior studies demonstrated that racial and ethnic minority groups in the United States experience disproportionately high rates of COVID-19–related hospitalization and in-hospital death (*2*). Examining the association between demographic factors and COVID-19 outcomes is pivotal to enhancing clinical decision-making, allocating resources, and prioritizing interventions.

Multiple studies have demonstrated differences in COVID-19 outcomes between racial minority groups and White persons. However, few studies have disaggregated minority groups into ethnic subgroups, particularly Asian, Native Hawaiian, and Pacific Islander groups (*2*). One study demonstrated that COVID-19 test positivity and hospitalization rates were highest in Filipino, Pacific Islander, and other Asian subgroups but did not find notable variation in intensive care unit (ICU) admission or death (*3*). Because socioeconomic determinants of health, such as income and education level (*4*), and underlying conditions, such as diabetes mellitus and obesity, differ greatly between those ethnic subgroups (*5*), further investigation of outcomes among those subgroups is warranted.

The population of Hawaii includes a higher percentage of ethnic groups that are often underrepresented in studies conducted in the continental United States, namely Native Hawaiian, Pacific Islander, and Asian American populations (*6*). Prior studies showed that those groups had disproportionately higher rates of COVID-19 than other groups in Hawaii and in the continental United States (*7*). Native Hawaiian and Pacific Islander populations also were found to have lower rates of COVID-19 vaccination than non-Hispanic White and Asian groups (*8*) and were underrepresented in studies regarding the uptake and efficacy of COVID-19 vaccinations (*9*). Comparing outcomes between vaccinated and unvaccinated persons in ethnic groups can provide valuable insights for shaping public health vaccination strategies and resource allocation within communities encompassing those populations. We investigated differences in outcomes among underrepresented racial and ethnic groups by reviewing COVID-19 fatality rates among hospitalized patients in a large tertiary care healthcare system in Hawaii, USA.

## Methods

We conducted a retrospective study of hospitalized patients with COVID-19 diagnosed via PCR and treated at a large tertiary care in-hospital provider in Hawaii during February 2020–August 2022. We collected deidentified data directly from the electronic medical record (EMR) of Queen’s Medical Center Health System, which includes 745 inpatient beds across 3 islands and is the main hospital on Oahu, serving as the quaternary referral center for the state of Hawaii.

We reviewed a total of 5,900 patient medical records; we excluded 406 records that were repeat admissions of individual patients, leaving 5,494 unique patients. As a part of the health system’s policy, all patients admitted for hospitalization were screened for SARS-CoV-2, and we included all hospitalized patients who tested positive within the study timeframe. We categorized patient age groups as 10–20, 21–30, 31–40, 41–50, 51–60, 61–70, and >71 years of age.

We calculated fatality rates by dividing the number of deaths by the total number of patients in a particular age or ethnic group, then multiplying by 100 to obtain a percentage. We investigated the association between death and race or ethnicity, age, sequential organ failure assessment (SOFA) score, and vaccination status in ICU and non-ICU settings.

We categorized patients by self-reported race or ethnicity according to the Hawaii Department of Health classifications: White, Native Hawaiian, Pacific Islander, Filipino, Japanese, Chinese, other Asian, Black, and other. The Pacific Islander group consisted primarily of persons who identified as Samoan. The other group included persons who identified as Puerto Rican, Mexican, Native American, Central American, Cambodian, Alaska Native, South American, Kosraean (persons from the Federated States of Micronesia), and persons who declined to respond. The other Asian group included persons who identified as Korean, Vietnamese, Laotian, Thai, Asian/East Indian, Middle Eastern, and other Asian groups.

The SOFA score (*10*) is a clinical score to assess a patient’s degree of organ failure on the basis of partial pressure of oxygen in blood, fraction of inspired oxygen, need for mechanical ventilation, platelet count, Glasgow coma score (*11*), serum bilirubin, mean arterial pressure, and serum creatinine. SOFA scores range from 0 to 24, and higher scores are associated with more severe illness and increased risk for death. For patients in this study, the EMR automatically calculated the SOFA score was if all parameters for SOFA were populated within patient records.

We defined vaccinated persons as those who received >1 of the available SARS-CoV-2 vaccinations before admission or during their hospital stay. Available vaccines were BNT162b2 (Pfizer-BioNTech, https://www.pfizer.com), Ad26.COV2.S (Johnson & Johnson/Janssen, https://www.janssen.com), and mRNA-1273 (Moderna, https://www.moderna.com). The deidentified dataset contained vaccine records adjudicated with the State of Hawaii COVID-19 vaccine registry. We quantified the total number of vaccinations regardless of the type of vaccine received.

To investigate the association between ethnicity and fatality rates, we performed age-specific regressions and stratified by vaccination status. We also performed regressions for the fatality rate and number of vaccinations received. In addition, we performed SOFA score–specific regressions of fatality rates to assess the association between SOFA score and death, and we adjusted for race or ethnicity and vaccination status.

## Results

**Figure 1:**
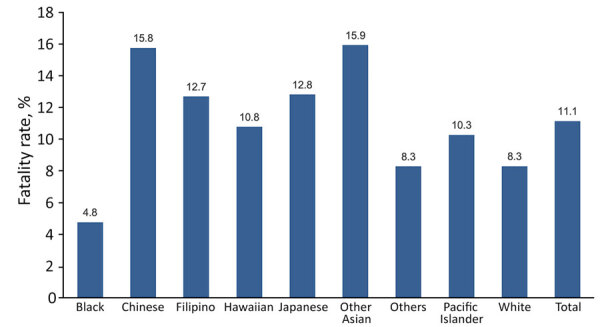
Overall fatality rates in study of differences in COVID-19 fatality rates among ethnic groups, Hawaii, USA, 2020–2022.

Of 5,494 patients included, 17% were Pacific Islander, 25% were Filipino, 13% were Native Hawaiian, 4% were other Asian, 5% were Chinese, 10% were Japanese, 20% were White, 2% were Black, and 3% were other (Table). Among our cohort, we noted 612 deaths, an overall fatality rate of 11.1%, of which Filipino persons comprised 28%, Pacific Islanders 16%, White persons 15%, Native Hawaiian persons 13%, Japanese persons 11%, Chinese persons 8%, other Asian persons 6%, other 2%, and Black persons 1% (Table). We noted the highest in-hospital fatality rates (15.9%) among persons of other Asian ethnicity, followed by Chinese (15.8%), Japanese (12.8%), Filipino (12.7%), Native Hawaiian (10.8%), Pacific Islander (10.3%), White (8.3%), Other (8.3%), and Black (4.8%) (Figure 1).

**Table:** Clinical characteristics and underlying conditions in a study of differences in COVID-19 fatality rates among ethnic groups, Hawaii, USA, 2020–2022*

Characteristics	Total	Race or ethnicity								
		Black	Chinese	Filipino	Native Hawaiian	Japanese	Other Asian	Other ethnicity	Pacific Islander	White
Total no.	5,494 (100)	105 (2)	292 (5)	1,379 (25)	733 (13)	523 (10)	226 (4)	181 (3)	935 (17)	1,120 (20)
Sex										
M	3,036 (55)	66 (63)	163 (56)	806 (58)	370 (51)	278 (53)	108 (48)	101 (56)	496 (53)	648 (58)
F	2,458 (45)	39 (37)	129 (44)	573 (42)	363 (49)	245 (47)	118 (52)	80 (44)	439 (47)	472 (42)
Mean age, y	59.3	50.9	64.3	62.5	54.2	69.8	60.4	52.3	52.1	60.1
No. deaths	612 (11)	5 (5)	46 (16)	175 (13)	79 (11)	67 (13)	36 (16)	15 (8)	96 (10)	93 (8)
Fatality rate, %†	11.1	4.8	15.8	12.7	10.8	12.8	15.9	8.3	10.3	8.3
Underlying conditions										
Diabetes	1,801 (33)	19 (18)	89 (31)	485 (35)	263 (36)	176 (34)	70 (31)	51 (28)	421 (45)	227 (20)
Hypertension	2,348 (43)	36 (34)	122 (42)	675 (49)	336 (46)	248 (47)	80 (35)	54 (30)	407 (44)	390 (35)
Atrial fibrillation	505 (9)	6 (6)	31 (11)	138 (10)	83 (11)	48 (9)	8 (4)	8 (4)	63 (7)	120 (11)
CHF	750 (14)	10 (10)	36 (12)	182 (13)	149 (20)	71 (14)	21 (9)	6 (3)	132 (14)	143 (13)
Obesity	1,974 (36)	34 (32)	70 (24)	315 (23)	381 (52)	101 (14)	36 (16)	61 (34)	596 (64)	380 (34)
Malignancy	43 (0.7)	2 (1.9)	2 (0.7)	8 (0.6)	6 (0.8)	8 (1.5)	2 (0.9)	1 (0.5)	4 (0.4)	10 (0.9)

*Values are no. (%) except as indicated. CHF, congestive heart failure. †Calculated by dividing the number of deaths by the total number of patients per group, then multiplying by 100. ‡Considered body mass index >30.

**Figure 2:**
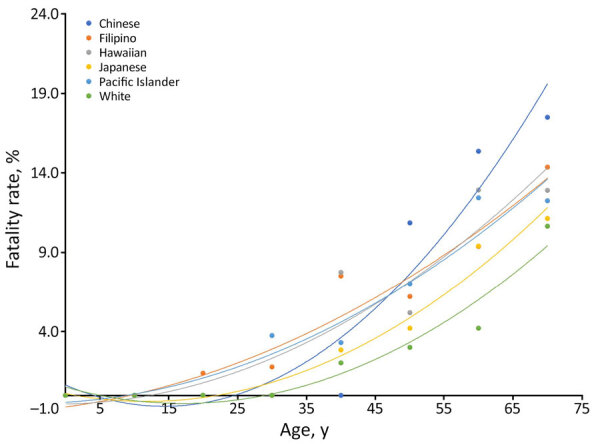
Fatality rates by age in study of differences in COVID-19 fatality rates among ethnic groups, Hawaii, USA, 2020–2022. Lines show polynomial trends among each racial or ethnic group; dots indicate average per 10-year age group. Black dotted line indicates mean fatality rate.

We found fatality rates were positively associated with increasing age across all racial and ethnic groups (Figure 2). Age-adjusted specific fatality rates were highest among Chinese persons and overlapped in Native Hawaiian, Filipino, and Pacific Islander persons. In the oldest age group, >71 years, Chinese persons had the highest fatality rates and White persons had the lowest.

**Figure 3:**
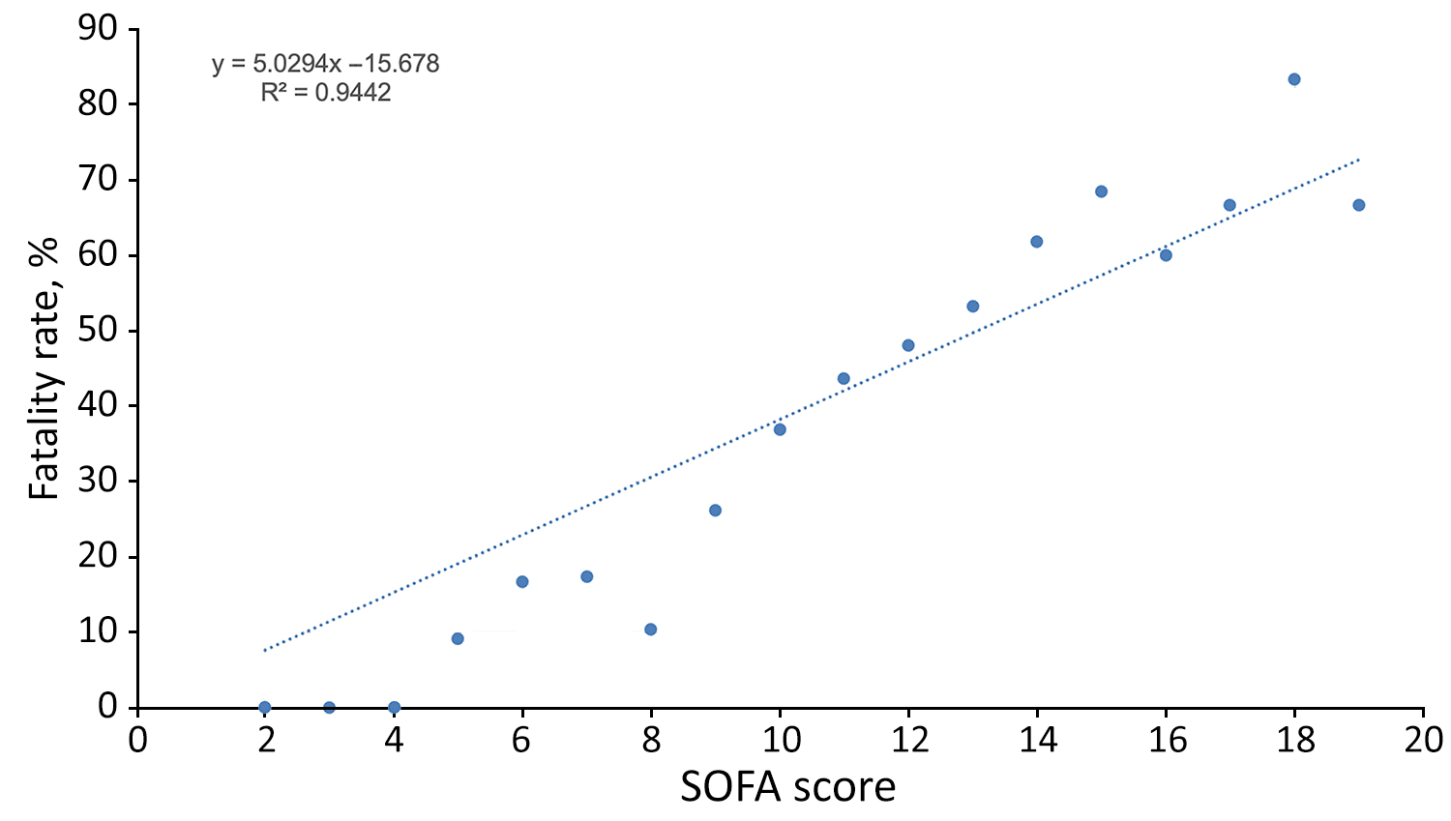
Correlation between SOFA score and death in a study of differences in COVID-19 fatality rates among ethnic groups, Hawaii, USA, 2020–2022. We used SOFA scores collected at admission for 562/5,494 (10.2%) patients included in the study. Dotted line indicates trend in fatality rate by SOFA score; dots indicate average fatality rate by SOFA score. We found higher SOFA scores strongly correlated with higher fatality rates. SOFA, sequential organ failure assessment.

We also found that SOFA scores were positively associated with fatality rates. However, only 562 (10.2%) patients had a SOFA score calculated at admission; most (89.8%) did not. Among the patients who had a SOFA score, mean scores were highest (12.26) among Chinese persons, followed by persons in the other Asian (11.96), Japanese (11.93), Native Hawaiian (11.76), Pacific Islander (11.57), Filipino (11.41), other (11.10), White (11.03), and Black (9.86) groups. However, the differences in mean SOFA score between ethnic groups were not statistically significant, and SOFA score at admission was linearly correlated with increasing fatality rates (R^2^ = 0.94; p<0.05) (Figure 3).

**Figure 4:**
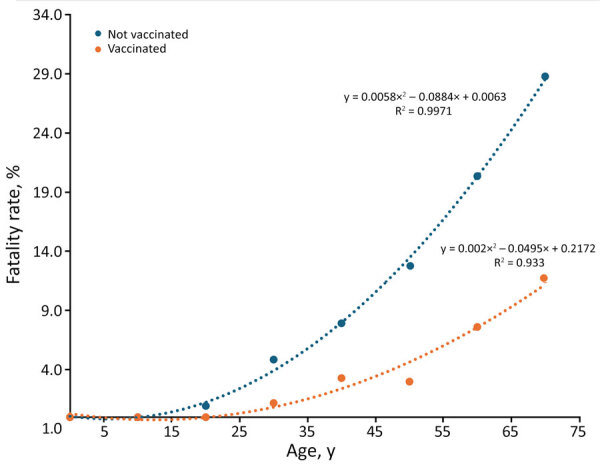
Fatality rates by vaccination status and age in a study of differences in COVID-19 fatality rates among ethnic groups, Hawaii, USA, 2020–2022. Dotted lines show trends among vaccinated (orange) versus unvaccinated (blue) patients; dots indicate average fatality rate per 10-year age group.

**Figure 5:**
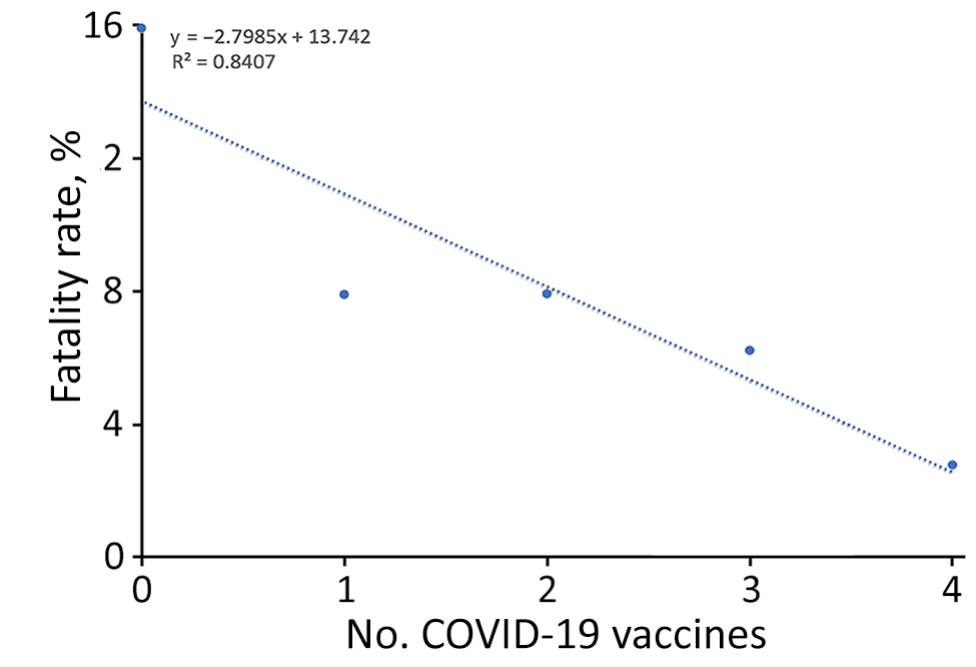
Fatality rates by vaccination status in a study of differences in COVID-19 fatality rates among ethnic groups, Hawaii, USA, 2020–2022. Dotted line show trends per number of COVID-19 vaccines received; dots indicate average fatality rate per vaccine group.

We found that unvaccinated patients in every age group had higher fatality rates than did vaccinated patients (Figure 4). Age-adjusted specific mortality rates in the unvaccinated group were higher than in the vaccinated group. Although fatality rates were positively associated with increasing age among vaccinated and unvaccinated groups, the fatality rate increased exponentially with age in the unvaccinated group compared with the vaccinated group. We found that the fatality rate was inversely associated with the number of COVID-19 vaccinations received, and an increased number of vaccinations correlated with lower fatality rate (R^2^ = 0.84) (Figure 5); that relationship was also true for patients who received mechanical ventilation. Although the fatality rate was higher in the group that received mechanical ventilation than the overall study sample, the linear regression coefficient was similar between that group and the overall study sample (R^2^ = 0.81 vs. 0.84; p<0.05). 

**Figure 6:**
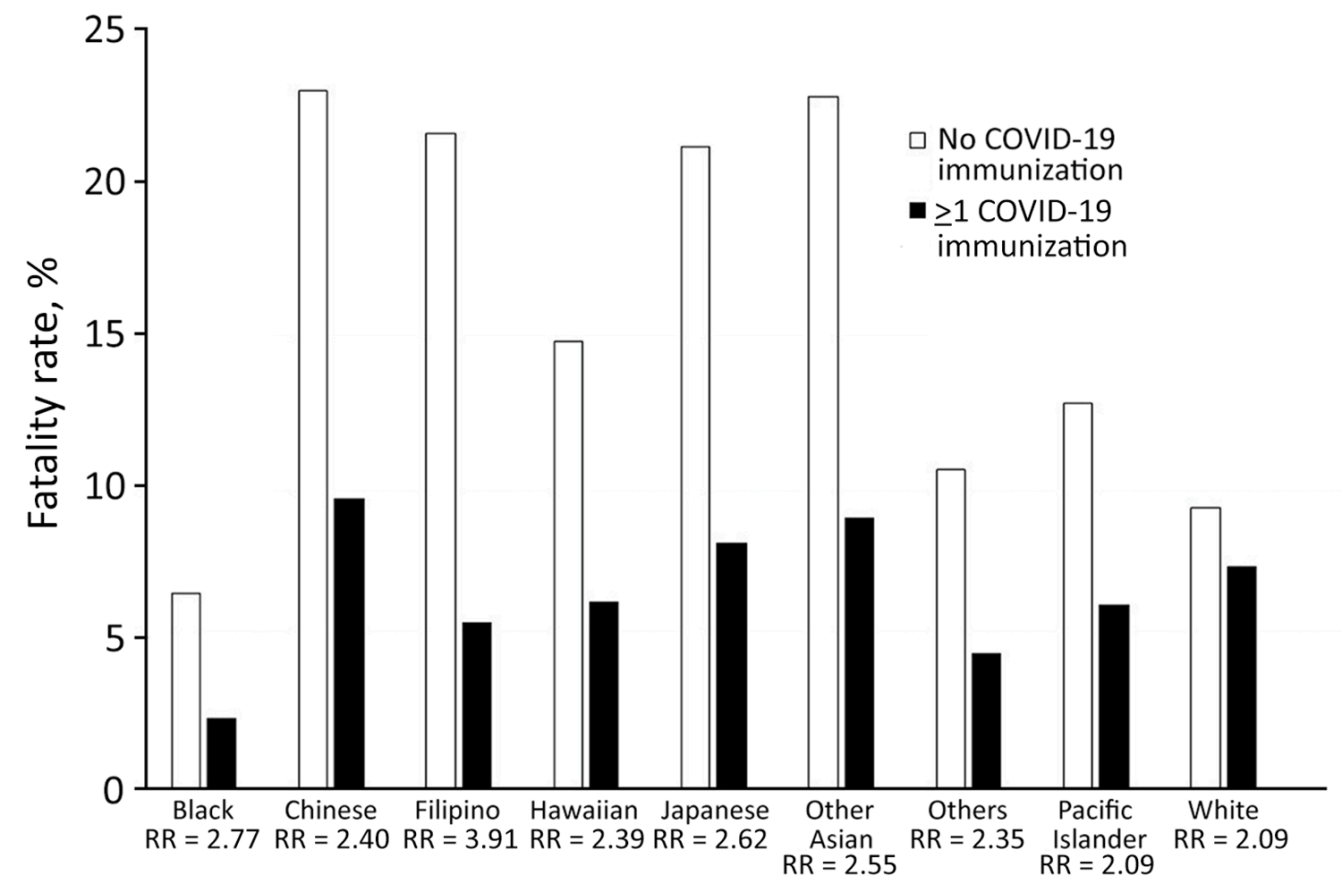
Fatality rates and RR by vaccination status in a study of differences in COVID-19 fatality rates among ethnic groups, Hawaii, USA, 2020–2022. Graph shows differences in fatality rates among racial and ethnic groups on the basis of vaccination status and overall RR for each group for persons vaccinated against COVID-19. RR, risk reduction.

Fatality rates were also higher in the unvaccinated group compared with the vaccinated group across all ethnicities. Vaccinated Filipino persons had the highest risk reduction (RR) at 3.91, followed by Black (RR = 2.77), Japanese (RR = 2.62), other Asian (RR = 2.55), Chinese (RR = 2.4), other (RR = 2.35), Pacific Islander (RR = 2.09), and White persons (RR = 1.27) (Figure 6).

## Discussion

We investigated in-hospital fatality rates in Hawaii, a state with a high percentage of Asian, Native Hawaiian, and Pacific Islander populations that are underrepresented in national COVID-19 research (*12*). We attempted to further disaggregate Asian and Pacific Islander populations into more specific subgroups of Japanese, Native Hawaiian, Filipino, Chinese, and other Asian groups. We found that the highest ICU fatality rates were among the Chinese and other Asian groups and that rates differed greatly between ICU and non-ICU settings. However, the total number of hospitalized persons from those groups was smaller than admissions among the Filipino, Native Hawaiian, and Pacific Islander populations. That finding is consistent with findings from previous studies in the United States and the United Kingdom that show higher ICU admission and fatality rates among Asian populations (*13*,*14*). However, our study also demonstrated that less-represented ethnic groups, including Native Hawaiian, Pacific Islander, and Filipino persons, also have higher in-hospital fatality rates than White persons. Despite the differences in race and ethnicity, overall hospital fatality rates in this study were lower than those in the continental United States (*15*). In addition, >95% of hospitalized patients in this study were insured, suggesting that difference in access to medical care is not the predominant factor in fatality rate differences.

The findings of our study are also aligned with that of previous studies showing that SOFA score is a reliable prognostic indicator of illness and death in COVID-19 patients (*16*,*17*). Although 89.8% of hospitalized patients in our study did not have a SOFA score, among those that did, we found that a SOFA score at admission was strongly correlated with fatality rate. We considered evaluating other scores, such as the Charlson Comorbidity Index (*18*), the COVID-19 severity index (*19*), and the MuLBSTA score for viral pneumonia mortality (*20*), to predict fatality rates among patients with diagnosed COVID-19. However, the Charlson Comorbidity Index is designed to predict long-term rather than acute death (*18*), and the MuLBSTA score and COVID-19 severity index do not incorporate many of the markers of systemic organ failure that are included in the SOFA score (*19*,*20*). Therefore, we concluded that the SOFA score, which is widely used in hospital settings, was a more appropriate tool for assessing fatality rates and accounting for underlying conditions in hospitalized COVID-19 patients. 

An analysis of variance showed that the average SOFA score did not differ greatly among ethnic groups despite substantial differences in fatality rates among groups. Further analyses also showed that differences in fatality rates between ethnic groups persisted even when adjusting for SOFA score, suggesting other health differences influenced fatality rates. Those findings suggest that differences in fatality rates between different racial and ethnic groups are influenced by additional ethnicity-related factors aside from SOFA score, echoing previous findings (*21*). We also found the association between SOFA score and fatality rate persisted when adjusted for age, vaccination status, and underlying conditions. A 2019 study measuring SOFA scores within racial differences in COVID-19 patients found that Black non-Hispanic patients were more likely than White non-Hispanic patients to have a SOFA score >6 (*22*). Other studies also suggest that higher SOFA scores are tied to increased mortality prediction rates and are higher than in non-White populations (*23*). Those findings suggest that widespread use of SOFA in clinical workflows could help care teams identify patients at higher risk for death during the admission process.

We found that fatality rates increased exponentially with age and decreased with confirmed vaccination status and increasing number of vaccinations. Those age-related findings are consistent with previous studies, such as the National Hospital Care Survey, which measured inpatient COVID-19 mortality rates during March 18, 2020–December 27, 2022, in 40 US hospitals (*15*,*24*). Those data showed consistently higher fatality rates for hospitalized patients >60 years of age compared with hospitalized patients 30–59 or 0–29 years of age. Our findings also align with findings from other studies that showed fatality rates among vaccinated hospitalized persons were lower worldwide (*25*,*26*). In our results, we also stratified vaccination status with age and found that fatality rates increased with both advancing age and lack of vaccination. Previous studies have shown that COVID-19 death rates among patients <65 years of age were higher among Hispanic and non-White non-Hispanic patients than in other ethnic groups (*27*).

Our data illustrate the benefits of COVID-19 vaccination across all racial and ethnic groups. However, we found that stratifying by ethnicity further elucidated differences in RR, showing that Filipino persons had the highest RR and White persons had the lowest. Paired with the disparate findings in fatality rates, those findings seem to suggest that lack of COVID-19 vaccination disproportionately affects certain minority populations. The benefits of COVID-19 vaccination can be profound, and Filipino, Native Hawaiian, and Pacific Islander populations that have the lowest vaccination rates in Hawaii could see the highest benefits. Lack of trust in providers and state government continues to be a major issue in getting those ethnic groups fully vaccinated (*28*).

Our findings can possibly be attributed to more underlying conditions in patients with advanced age, non-White ethnicities, or societal inequalities influencing social determinants of health. As has been established by previous research, racial and ethnic minority groups experienced the brunt of the COVID-19 pandemic in higher hospitalization and fatality rates (*29*).

Our study also shows that increased number of vaccinations is associated with decreased fatality rates, which aligns with studies showing decreased fatality among persons who had at least a 2-dose vaccination series with or without a COVID-19 booster (*30*–*32*). Furthermore, our study showed that vaccination nearly equalized the fatality rate across racial and ethnic groups, and those groups also had similar RR. Those results emphasize the critical role of vaccination in preventing severe illness and speak to the comparable efficacy of the available vaccinations across ethnic groups, all of which support the benefit of continued COVID-19 vaccination even in the face of vaccine hesitancy.

The first limitation of this study is the complex and multifaceted reasons for differences in illness and death between ethnic groups. Although socioeconomic factors, including limited access to healthcare, higher prevalence of underlying health conditions, and disparities in social determinants of health, can play a role, we did not examine nor adjust for those factors in our study (*13*,*14*,*33*). Recognizing and addressing those differences is crucial in working toward equitable health outcomes. The second limitation is that the study included patients hospitalized with COVID-19 as their primary diagnosis and those who were incidentally diagnosed with COVID-19 after admission; thus, many patients could have had other acute medical conditions associated with illness and death. Third, all data used in this study was collected from a single hospital system exclusive to the state of Hawaii (albeit the largest hospital system in Hawaii) and therefore are not generalizable to the country. In addition, sample sizes for some groups within study (for example, Black) were relatively small and might not reliably reflect the broader populations from which they were derived. Finally, SOFA scores were only calculated in patients for whom all SOFA score parameters were populated within the EMR. Therefore, all analyses on the basis of SOFA score include only a small percentage of the overall study sample, which might introduce selection bias.

Despite its limitations, this study included a diverse sample, extended timeframe, and objective confirmation of COVID-19 diagnosis. The diverse population represented in this study enabled analysis of COVID-19 outcomes in ethnic groups that are underrepresented in national data. The study also featured >2 years of data and spanned multiple SARS-CoV-2 variants, accounting for discrepancies in outcomes between variants that have been shown to have different rates of hospitalization and death (*34*,*35*). In addition, confirmation of COVID-19 diagnosis via standardized hospital PCR testing minimizes interference from differing accuracy between multiple testing modalities. Overall, our study was robust in showing that fatality rates in COVID-19 patients differed by ethnicity, age, SOFA score, and vaccination status.

In conclusion, we found that Chinese, Japanese, Filipino, Native Hawaiian, and Pacific Islander patients with COVID-19 had higher in-hospital fatality rates than White patients. Our investigation revealed a strong positive correlation between in-hospital fatality rate and age, consistent with the existing literature. In addition, we determined that SOFA score was predictive of in-hospital death across all racial and ethnic groups. Our findings reaffirmed the association between COVID-19 vaccination and decreased in-hospital fatality rates across various racial and ethnic groups and that risk for in-hospital fatality decreased as number of COVID-19 vaccinations increased. Clinicians should consider those factors when treating COVID-19 patients of those racial and ethnic groups.
